# Supplementary material for: Systematic characterization of cell type-specific master metabolic regulators in Alzheimer’s disease
Source: Res Sq. 2025 Aug 18:rs.3.rs-7207381. Preprint. [Version 1] doi: 10.21203/rs.3.rs-7207381/v1 (PMC12393479; doi:10.21203/rs.3.rs-7207381/v1)
Supplement: 1 [file NIHPPRS7207381V1-supplement-1.pdf]

Supplementary Figure Legends

Extended Data Fig. 1 | Profiling cell-specific metabolic heterogeneity in AD in the DLPFC. (a) Pathway activity scores for metabolic pathways across cell types in the DLPFC region. Differences between AD and non-AD that are greater than 0.1 indicates significant changes. Orange indicates up-regulated pathways in AD, while green indicates the pathways that are down-regulated in AD. (**b)** Summary of the number of significant metabolic pathways in AD as illustrated in **a**. (**c)** Exemplar metabolic pathways in InN and microglia. Fig. colors and labels format are consistent with Fig. 2.

Extended Data Fig. 2 | Summary of cell type-specific metabolite-sensor pairs from scFUMES. (a) Summary of metabolite-sensor physical/functional measurements retrieved from ChEMBL and BindingDB databases. (**b-c)** Categories of metabolites and targets from (a). **(d)** The numbers of pairs, targets and metabolites per cell types. **(e-f)** Target analysis across different cell types in AD (**e**) or non-AD (**f**). The targets that have

been reported to associate with AD are denoted with “*”. The target numbers per each cell type are labeled in parentheses. The colors represent the number of metabolites for each target. (**g-h)** Exemplar 3D complex structures of FABP3-palmitic acid (**g,** PDB ID: 4TKJ) specific in AD ExN cells and FFAR3-butyric acid specific in non-AD ODC cells (**h,** PDB ID: 8J21).

Extended Data Fig.3 | Gene expression alterations of selected proteins in different human brain regions and cell types. (a) FABP3 expression changes in brain regions (STG, PHG and IFG) at the transcriptomics RNA-seq level (AD versus non-AD). (**b)** Gene expression alterations of FABP3 in ExN, VDR in microglia and FFAR3 in ODC. Log2Foldchange (log_2FC) were calculated compared AD to non-AD. Each point represents one individual.

Extended Data Fig.4 | The functional effects of scFUMES-predicted FABP3 or KYAT1 on pTau-231. Western blot and quantitative analysis showing the overexpression of scFUMES-predicted FABP3 or KYAT1 significantly reduced insoluble pTau-231 (p = 5 x 10^{-4} for FABP3, p = 0.041 for KYAT1, n =3) while not soluble pTau-231 (p = 0.056 for FABP3, p = 0.82 for KYAT1, n =3 replicates). If not stated otherwise, data are the mean ± s.e.m. Unpaired Student’s t-test. *, p < 0.05; **, p < 0.01; ***, p < 0.001; ****, p < 0.0001; ns, not significant.

Extended Data Fig.5 | Summary of genetics-supported AD likely causal metabolites. Continue to **Fig. 4**. We predicted AD likely causal metabolite based on

four latest metabolite GWAS and three AD GWAS datasets were employed. Four MR methods were adopted, including IVW, MaxLik, MRpresso and Weighted Median. Significance was determined with FDR < 0.05.

Extended Data Fig.6 | Metabolic pathway analysis for AD-associated metabolites from the metabolomics studies. Significant metabolites in AD were denoted in different colors (FDR < 0.05). Related metabolic pathways were connected to specific metabolites. Metabolic pathways were also seen in **Fig. 2c**.

Extended Data Fig. 7 | The protein level changes of KYAT1 knockdown in vitro. knockdown of KYAT1 via siRNA significantly reduce the KYAT1 protein level ($p < 10^{-4}$). If not stated otherwise, data are the mean ± s.e.m. Unpaired Student's t-test. *, $p < 0.05$; **, $p < 0.01$; ***, $p < 0.001$; ****, $p < 0.0001$; ns, not significant.

Extended Data Fig. 8 | Gene expression alterations of KYAT1 in single cell or transcriptomic level. Differential expression of KYAT1 in FP (in male), PCC brain regions at bulk RNA-seq level and in ExN at single-cell level.

Extended Data Fig. 9 | Genetic-supported metabolites-mediated metabolite-sensor network by AD risk factors in the MTG. (a) Network depicting sex-specific metabolite-sensor pairs. Line colors indicate the female or male-specific pairs. Metabolites are shown in circle, and proteins are shown in square. MR-prioritized metabolites and their pairs were labeled. (**b)** Histogram showing number of cell-specific

genetic-supported metabolite-sensor pairs within female or male samples. **(c)** Microglia specific genetic-supported metabolite-sensor pairs. (**d)** Network depicting metabolite-sensor pairs in samples with *APOE4* or non-*APOE4* phenotypes. Line colors indicate the *APOE4* or non-*APOE4*-specific pairs. (**e)** Histogram showing number of cell-specific genetic-supported metabolite-sensor pairs within *APOE4* or non-*APOE4* samples. (**f)** Microglia specific genetic-supported metabolite-sensor pairs.

Extended Data Fig. 10 | Genetic-supported metabolites-mediated metabolite-sensor network by AD risk factors in the DLPFC. (a-b) Network depicting sex-specific metabolite-sensor pairs (**a**) and APOE4-specific pairs (**b**). Line colors indicate the sex-specific or APOE4-specific pairs. Metabolites are shown in circle and proteins are shown in square. MR-prioritized metabolites and their pairs were labeled. The colors or labels styles are consistent with **Fig. 6**.

Extended Data Fig. 11 | Differences of genetic-supported metabolites-mediated metabolite-sensor network across AD risk factors. (a-b) Network representations of sex-specific (**a**) or APOE4-specific (**b**) AD likely causal pairs in both MTG and DLPFC regions.

Supplementary Files

This is a list of supplementary files associated with this preprint. Click to download.

- ExtendedDataFig1.pdf
- ExtendedDataFig2.pdf
- ExtendedDataFig3.pdf
- ExtendedDataFig4.pdf
- ExtendedDataFig5.pdf
- ExtendedDataFig6.pdf
- ExtendedDataFig7.pdf
- ExtendedDataFig8.pdf
- ExtendedDataFig9.pdf
- ExtendedDataFig10.pdf
- ExtendedDataFig11.pdf
- SupplementaryFigureLegends.pdf
